# Therapeutic Vaccine Strategies against Human Papillomavirus

**DOI:** 10.3390/vaccines2020422

**Published:** 2014-06-13

**Authors:** Hadeel Khallouf, Agnieszka K. Grabowska, Angelika B. Riemer

**Affiliations:** Immunotherapy and -prevention, German Cancer Research Center (DKFZ), Heidelberg 69120, Germany; E-Mails: h.khallouf@dkfz.de (H.K.); a.grabowska@dkfz.de (A.K.G.)

**Keywords:** human papillomavirus (HPV), therapeutic vaccination, cancer immunotherapy, epitopes, cytotoxic T cells (CTL), T helper cells (Th), cervical cancer, peptide vaccination, nanoparticles (NPs), DNA vaccination, dendritic cell (DC) vaccination, vector-based vaccination, adjuvants

## Abstract

High-risk types of human papillomavirus (HPV) cause over 500,000 cervical, anogenital and oropharyngeal cancer cases per year. The transforming potential of HPVs is mediated by viral oncoproteins. These are essential for the induction and maintenance of the malignant phenotype. Thus, HPV-mediated malignancies pose the unique opportunity in cancer vaccination to target immunologically foreign epitopes. Therapeutic HPV vaccination is therefore an ideal scenario for proof-of-concept studies of cancer immunotherapy. This is reflected by the fact that a multitude of approaches has been utilized in therapeutic HPV vaccination design: protein and peptide vaccination, DNA vaccination, nanoparticle- and cell-based vaccines, and live viral and bacterial vectors. This review provides a comprehensive overview of completed and ongoing clinical trials in therapeutic HPV vaccination (summarized in tables), and also highlights selected promising preclinical studies. Special emphasis is given to adjuvant science and the potential impact of novel developments in vaccinology research, such as combination therapies to overcome tumor immune suppression, the use of novel materials and mouse models, as well as systems vaccinology and immunogenetics approaches.

## 1. Introduction

Cervical cancer is the third most common cancer worldwide [1,2]. Annually, almost half a million women are diagnosed with cervical cancer [3]. Over 80% of all cervical cancer cases occur in developing countries [4]. Cervical cancer and its precursors are caused by various types of the human papillomavirus (HPV) [5]. The HPV family comprises over 170 types that are classified as low-risk (LR), high-risk (HR), and potentially/probably HR in terms of their oncogenic potential [6]. Around 20 of these are involved in anogenital and oropharyngeal squamous cell carcinoma development, and are transmitted during sexual activity [7,8,9,10].

Two prophylactic HPV vaccines are currently available. The bivalent vaccine protects against the two HR-HPV types 16 and 18; the quadrivalent vaccine protects against infection with HPV16/18 and also against the LR types 6 and 11 (causing genital warts). Unfortunately, vaccine coverage as yet is not optimal in many countries. Possible obstacles are cost and socio-cultural factors. Moreover, cancers can also be caused by the spectrum of non-16/18 HR types that are not covered by the currently existing vaccines (around 30% of cases in cervical cancer [11]). Increasing numbers of potentially/probable HR HPV are being found in anogenital lesions, especially in human immunodeficiency virus (HIV) infected subjects [12]. Furthermore, the available preventative vaccines have no therapeutic effects, *i.e.*, they are not effective once HPV infection is established. The time between viral infection and tumor development is 10–20 years in persons having developed a persistent infection, so a large proportion of the global population is already infected and cannot be treated by the existing vaccines [13]. Existing therapeutic modalities for HPV-induced premalignant lesions are surgical, and can lead to impaired function of the affected tissue (such as causing premature births in pregnancies following cervical conizations). Thus, it is of great importance to develop novel non-invasive treatment strategies, among which therapeutic HPV vaccines are an attractive option [14,15].

## 2. Current Therapeutic HPV Vaccine Strategies

### 2.1. Importance of Choosing the Appropriate Antigen

Among the eight HPV proteins, E2, E5, E6, and E7 are regarded as being crucial for HPV immune escape and malignant progression. E2 and E5 are expressed soon after infection, prompting HPV immune escape mechanisms and initiating carcinogenic progression [16,17,18,19]. E6 and E7 are the major transforming proteins. The E7 protein binds to the retinoblastoma gene product, pRb [20], while the E6 protein interacts with the p53 tumor suppressor protein [21], leading to their degradation. E6 and E7 are constitutively expressed in both premalignant and advanced lesions, making them ideal targets for immunotherapeutic approaches for HPV-induced malignancies [22,23]. More recently, E2 and E5-targeted approaches have also been investigated [19]. Almost all therapeutic HPV studies to date have been conducted with antigens from the most abundant HR type, HPV16. Due to concerns about applying whole oncogenes/proteins in humans, most studies have used versions of E6/E7 that have been mutated in order to abrogate p53/pRb binding, respectively.

### 2.2. Protein-Based Therapeutic HPV Vaccines

The oncoproteins E6 and E7 have been extensively used in early therapeutic HPV vaccine studies. E7 alone has been used more frequently than E6 due to the fact that it is more abundantly expressed and is more highly conserved than the E6 protein [24]. E7 is a small protein which is poorly immunogenic (11 kDa). Hence it was used with immunological adjuvants, and immunogenic protein carriers that enhance antigen delivery, such as heat shock proteins (Hsp’s) or bacterial proteins (e.g., adenylate cyclase (CyaA) and certain toxins, see below). Various forms of therapeutic vaccines based on E7 have been developed and tested in **animal models**. Most of the vaccines induced E7-specific cytotoxic T cell (CTL) responses and were effectively inducing tumor regression in animal models of HPV16 tumors. Some recent examples include HPV16 E7 fused to HBcAg and Hsp65 [25], HPV16 E7 fused to the CyaA of *Bordetella pertussis* [26], or HPV16 E7 fused to a bacterial lipid moiety to form a lipoprotein vaccine [27]. Fusion protein vaccines targeting both E6 and E7 have also been investigated. A recent animal study showed that an E6-E7 fusion protein (linked to exotoxin A domains of *Pseudomonas aeruginosa*) generated both stronger E6 and E7-specific immunity and anti-tumor effects than either antigen alone [28]. Nevertheless, only few protein-based vaccines have reached the **clinical trial phase** [29,30,31,32,33,34,35,36,37,38,39,40,41] (listed in Table 1 and reviewed in [42,43,44,45]). Complete responses in phase II trials ranged between 35% in 21 patients with anogenital intraepithelial neoplasia [39] and 22% in 58 cervical intraepithelial neoplasia grade 3 (CIN3) patients [40]. These two studies used HspE7 (a fusion protein consisting of HPV16 E7 and Hsp65 from *Mycobacterium bovis*, also called SGN-00101). However, it was unclear whether the response mediated by HspE7 was due to natural regression rather than treatment effects. HspE7, which targets the HPV16 E7 oncoprotein, also showed efficacy in patients infected with HPV types other than 16, suggesting cross-reactivity [40].

Of note, a fusion protein of HPV16 E6/E7 and L2 (TA-CIN) was tested in phase II clinical trials in patients with anogenital and vulvar intraepithelial neoplasia (VIN), comparing its effectiveness with HPV16 and 18 E6 and E7 encoded in a vaccinia virus vector (TA-HPV, discussed in section *Viral vector vaccines*) in heterologous prime/boost regimens. Neither TA-CIN alone nor any prime/boost combination offered advantages over single-agent TA-HPV. However, it was tested again in combination with the TLR7-agonist imiquimod in a clinical phase II study in 19 women with VIN2/3. Complete histologic regression of VIN2/3 was observed in 32% of patients at week 10 post-vaccination, increasing to 58% at week 20, and 63% at week 52 [30].

A separate project, not targeting malignancies but HPV6-mediated genital warts, also reached the clinical trial phase (phase II/III). Close to 300 patients were vaccinated with an HPV6 L2-E7 fusion protein. All showed specific antibody induction, but unfortunately no difference in wart recurrences was observed between the vaccine and placebo groups [41].

**Table 1:** Clinical studies with protein-based vaccines.

Antigen/Composition	Adjuvant	Route of Injection	Phase of Study	Patient Population	Immune Response	Clinical Response	References
HPV16 E7 + adenylate cyclase (ProCervix)	Imiquimod	ID	I	47 HPV16^+^ and/or 18^+^ women with normal cervical cytology	Antigen-specific T cell responses	High viral clearance	PC10VAC01
ProCervix	Imiquimod	ID	II	220 HPV16^+^ and/or 18^+^ women with normal cervical cytology or mild cervical cellular dyskaryosis	Ongoing	Ongoing	PC10VAC02 NCT01957878
HPV16 E6 E7 and L2 fusion protein (TA-CIN)	-	IM	I	40 healthy volunteers	Antigen-specific T cell responses	No clinical measures in study	de Jong 2002 [29]
TA-CIN	Imiquimod	IM	II	19 VIN 2/3	CTL responses	12/19 CR	Daayana 2010 [30]
TA-CIN (prime) TA-HPV (boost)	-	IM ID	I	29 AGIN	Antigen-specific antibody/CTL responses	6/29 PR 19/29 SD	Smyth 2004 [31]
TA-HPV (prime) TA-CIN (boost)	-	ID IM	I	10 VIN 2/3	9/10 Antigen-specific antibody/CTL responses	3/10 PR	Davidson 2004 [32]
TA-CIN (prime) TA-HPV (boost)	-	IM ID	II	27 VIN 3 2 VAIN 3	Antigen-specific antibody/CTL responses	1/27 CR 5/27 PR 15/27 symptomatic improvement	Fiander 2006 [33]
HPV16 E6 and E7 recombinant bacterial fusion protein	ISCOMATRIX	IM	I	8 CIN 1 10 CIN 2 13 CIN 3	Antigen-specific antibody, T cell and DTH responses	4/31 CR (3 CIN 1 1 CINI2/3) 14 reduced viral load	Frazer 2004 [34]
HPV16 E7 (mutated protein) and protein D of *H. influenzae*-fusion protein	AS02B	IM	I/II	2 CIN 1 5 CIN 3	5/7 Antigen-specific T cell responses	CIN 3 0/5 PR CIN 1 2/2 PR	Hallez 2004 [35]
HPV16 E7 and Hsp65 fusion protein (HspE7)	-	SC	II	22 anal HSIL 14/22 anogential warts	None	3/14 CR 10/14 PR	Goldstone 2002 [36]
HspE7	-	SC	I/II	27 with respiratory papillomatosis	Not reported	Increase in median intersurgical interval	Derkay 2005 [37]
HspE7	-	SC	I/II	15 HIV^+^ AIN 2/AIN 3	Not reported	1/15 CR 4/15 PR 10/15 NR	Palefsky 2006 [38]
HspE7	-	SC	II	21 LSIL, HSIL, ASCUS or AGUS	9/17 Antigen-specific T cell responses	7/20 CR 1/20 PR 11/20 SD 1/20 NR	Roman 2007 [39]
HspE7	-	SC	II	58 CIN 3	Not reported	13/58 CR 32/58 PR 11/58 SD 2/58 NR	Einstein 2007 [40]
HPV6 L2-E7 fusion protein	AS02A	IM	II/III	320 with anogenital warts	Antigen-specific antibody response	NR	Vandepapelière 2005 [41]

Abbreviations: Hsp, heat shock protein; ID, intradermal; IM, intramuscular; SC, subcutaneous; VIN, vulvar intraepithelial neoplasia; VAIN, vaginal intraepithelial neoplasia; CIN, cervical intraepithelial neoplasia; AGIN, anogenital intraepithelial neoplasia; AIN, anal intraepithelial neoplasia; LSIL, low-grade squamous intraepithelial lesion; HSIL, high-grade intraepithelial lesion; ASCUS, atypical squamous cells of undetermined significance; AGUS, atypical glandular cells of uncertain significance; CTL, cytotoxic T lymphocyte; DTH, delayed type hypersensitivity; CR, complete response; PR, partial response; SD, stable disease; NR, no response; TA-HPV, recombinant vaccinia virus encoding HPV 16 and 18 E6/E7.

### 2.3. Peptide Vaccines

Synthetic peptides offer several advantages over whole proteins, particularly with regards to safety and ease of production. However, as with all subunit vaccines, special care must be taken to potently stimulate T cells and elicit immunological memory. Adjuvant use, lipopeptide conjugation and direct delivery to dendritic cells (DCs) are some of the approaches currently employed to overcome these problems. Synthetic peptides used in vaccination studies can be grouped in two categories: Synthetic long peptides (SLPs) and specific epitope (short) peptides. Long peptides potentially harbor CD8^+^ CTL and CD4^+^ T helper cell (Th) epitopes, whereas short peptides usually encompass only a single defined CTL epitope.

#### 2.3.1. Synthetic Long Peptides

Synthetic long peptides of the E6 and E7 oncoproteins are overlapping peptides of 15–35 amino acids that cover the entire sequence of the native protein antigens. They require internalization and processing by antigen presenting cells (APCs), such as DCs for presentation on major histocompatibility complex (MHC) class I and class II molecules [46]. Therefore, they do not require patients' human leukocyte antigen (HLA) typing before vaccination, as *in vivo* antigen processing ensures epitope selection based on each patient’s HLA profile. Furthermore, SLPs were found to facilitate simultaneous priming of T cells against multiple dominant and subdominant epitopes stimulating a broad T-cell response [47]. SLPs were thoroughly tested in **experimental models**, leading the way to clinical translation (e.g., [48], reviewed in [46]). Several long peptide-based therapeutic HPV vaccines have been tested in **clinical trials** (listed in Table 2). All of these pioneering studies have been conducted at the Leiden University Medical Center ([49,50,51,52,53,54], reviewed in [14]). A major breakthrough for the whole cancer vaccination field was the observation of a durable and complete regression in 47% of VIN3 patients treated with a HPV16 E6 and E7 SLP vaccine [51]. Clinical responses were associated with strong and broad HPV-specific CTL and Th type 1 (Th1) responses that peaked after the first vaccination [47,51]. Furthermore, the injection of HPV16 SLP induced strong HPV16-specific Th1 immunity in cervical cancer patients [49]. This was however without clinical success [53]. Despite the latter results, these studies demonstrated that these highly immunogenic vaccines are safe and capable of inducing the desired immune responses. The authors argue that for cancer vaccination to become clinically successful, combination with other therapies, which target regulatory mechanisms and local immunosuppression in the tumor microenvironment, might be necessary [45,55].

#### 2.3.2. Epitope-Specific Short Peptides

The use of longer peptides has also been discussed critically, as administration of a peptide fragment taken out of context of the whole antigen can result in unwanted immune responses. This is due to the fact that an exogenously administered peptide will not necessarily follow the same pathway of processing as the native pathogen or the cancer-cell derived antigen, and consequently might not elicit efficient anti-cancer immune responses [56]. An approach to ensure precisely targeted CTL responses is immunization with specific epitopes. It has long been known that linear short peptide epitopes composed of 8–11 amino acids are capable of inducing cytotoxic responses. However, the use of epitope-based vaccines is restricted to patients of a given HLA type, and as such requires HLA typing of prospective vaccinees before vaccine application.

HLA-class I restricted CTL epitopes have classically been defined by assays with overlapping short peptides that cover the whole sequence of the target antigen. HPV16 epitopes have also been determined in this way [57]. Currently, epitopes for a specific HLA molecule are mostly determined by prediction servers, which are based on specific binding motifs, taking into account so-called anchor residues. These are defined amino acids at specific positions within the peptide, characteristic for each HLA molecule. Lists of motifs are conveniently web based (e.g., SYFPEITHI, a prediction server and database of MHC ligands and peptide motifs [58], or the NetMHC family of prediction servers [59]). However, the success rate for *de novo* prediction of T-cell epitopes, even for well-studied and abundant MHC alleles, is only about 60% for many alleles and for new alleles or MHC I molecules from poorly studied ethnic populations no binding motifs are available. Recent studies have substantially improved the predictive capacity of algorithms for some well-studied alleles [60,61,62], however, it is still necessary to confirm HLA binding of a given predicted peptide experimentally. Further complications arise due to the paucity if immunodominant peptides which are selected from the numerous potential HLA ligands of a given pathogen [45]. Thus, predictive markers of immunogenicity must take into account not only peptide binding but also the abundance and density of the antigen that is present on the cell surface; the time of expression of the antigen during the infection or pathological process; the correct processing and luminal transport of the epitope; and the available T-cell repertoire in the host organism. Nevertheless, the participation of only a few epitopes in effective immunity limits the number of distinct epitopes that are required in a peptide-based vaccine to elicit a protective immune response.

Another opportunity to reduce the number of required epitopes lies in the exploitation of HLA supertypes. HLA supertypes are groups of HLA molecules that share peptide-binding specificity and therefore epitope presentation [63]. Thus, supertype motifs allow for a significant reduction in the number of epitopes required to give broad population coverage for a given pathogen. However, it should be noted that supertypes are not always predictive of stable peptide binding and significant variations, even between closely related alleles, can occur [64,65,66].

##### 2.3.2.1. CTL Epitope Detection by Mass Spectrometry

As outlined above, it is important to determine the true presence of a candidate epitope on the target cell. Bioinformatic approaches and HLA binding assays cannot predict, however, which peptides are actually processed and presented on the cell surface. Mass spectrometry (MS) techniques have been developed to directly assess the physical presence of CTL and Th epitopes on tumors (reviewed in [67]). For HPV-derived HLA class I epitopes, which are of low abundance due to viral immune evasion mechanisms, a special MS^3^ mass spectrometry approach has been devised, allowing highly sensitive detection of predicted target peptides on the cell surface. This method achieves sensitivities comparable with that of a T cell with a dynamic range of one peptide among 100,000 HLA molecules displayed per cell. It has been shown that, among E6 and E7-derived peptides, only a single 9-mer epitope was found on all HLA-A*0201 HPV-16-transformed epithelial tumor cells tested. This conserved peptide, E7_11–19_, was predicted to have the capacity to bind to the vast majority of globally distributed A2 alleles (100 of 116 HLA-A2 alleles) [68]. We are currently using this approach to identify further epitope candidates to be included in therapeutic HPV vaccine design.

##### 2.3.2.2. Identification of Promiscuous T Helper Cell Epitopes

Exclusive targeting of HLA class I-restricted HPV epitopes, without involving specific T cell help, can lead to suboptimal and short-lasting CTL responses. Since Th cells have been shown to be crucial for the induction and maintenance of CTL responses [69], and more recently to also be important for direct anti-tumor immunity [70], HLA class II-restricted epitopes are intensively investigated with the aim of improving the efficacy of HPV targeted immunotherapy. Most studies on identification of HPV-specific Th epitopes to date have focused on E6 and E7 as target antigens and overlapping peptide pools have been used [71,72,73,74,75]. Although successful, these approaches of systematic T cell epitope mapping are costly and time-consuming, as they require synthesis and several rounds of screening of peptides spanning the full length of the target antigen. Therefore, also for HLA class II epitopes, *in silico* prediction methods have been developed [76,77]. These allow prediction of epitopes “promiscuously” binding to multiple HLA class II alleles. Combination of these predictions with *ex vivo* immunological evaluation of epitope-specific Th immunity resulted in the identification of Th epitope vaccine candidates, which may improve the immune potency of CTL epitope-specific vaccination approaches [78].

##### 2.3.2.3. Current Epitope-Specific Vaccine Approaches

Several epitope-specific therapeutic HPV vaccines have been tested in **clinical trials** (listed in Table 2). These studies used different epitopes such as E7_11–20_, E7_12–20_ and E7_86-93_, either as non-modified peptides with different adjuvants [79,80], or as lipo-peptides [81,82]. However, the clinical responses in all these trials were modest, the best observed outcome being 18% complete responses and 50% partial responses in high-grade CIN or VIN patients [82]. Recently, a new delivery system (very small size proteoliposomes) was tested to deliver the HPV16-E7_86–93_ peptide and showed promising clinical responses in CIN2/3 patients [83]. Two ongoing large phase I studies are using novel adjuvants, such as the yeast extract Candin^®^ (NCT01653249), or HPV16 peptides combined with GM-CSF and Montanide ISA 51 (NCT00257738).

A promising **preclinical** study using a new adjuvant system (VacciMax^®^ liposomes) and a mix of HPV16 peptides E7_11–20_, E7_82–90_, E7_86–93_ and E6_29–38_ showed strong CTL responses induced by the vaccine in addition to complete eradication of TC-1 tumors (a murine tumor cell line transformed by *ras*, HPV16 E6 and HPV16 E7) [84]. Combining an E7 derived peptide with Hsp110 also induced strong immune and anti-cancer responses in the TC-1 tumor model [85].

These new vaccines still need to be evaluated in (larger) clinical trials; however they highlight the importance of using innovative adjuvants, which could significantly increase not only immunological but also clinical responses to the vaccines.

**Table 2:** Clinical studies with peptide-based vaccines.

Antigen/Composition	Adjuvant	Route of Injection	Phase of Study	Patient Population	Immune Response	Clinical Response	References
Overlapping synthetic long peptides from HPV16 (9 E6 and 4 E7) (HPV16-SLP)	Montanide ISA 51	SC	I	35 end-stage CxCa	CTL response	NR	Kenter 2008 [49]
HPV16-SLP	Montanide ISA 51	SC	II	6 with resected CxCa	CTL response	NR	Welters 2008 [50]
HPV16-SLP	Montanide ISA 51	SC	II	20 VIN 3	CTL response	9 CR 6 PR	Kenter 2009 [51]
HPV16-SLP	Montanide ISA 51	SC	II	9 HSIL	CTL response	NR	de Vos van Steenwijk 2012 [52]
HPV16-SLP	Montanide ISA 51	SC	II	20 with advanced or recurrent gynecological carcinoma	CTL response	NR	van Poelgeest 2013 [53]
HPV16-SLP	Montanide ISA 51	SC	II	50 with LSIL or persistent mild cytological cervical abnormalities	Antigen-specific CTL response	NR	de Vos van Steenwijk 2014 [54]
HPV16 E7_11-20_, E7_86-93_ and PADRE	IFA	SC	I/II	19 recurrent or residual CxCa	No antigen-specific CTL response	2/19 PR 2/19 SD	van Driel 1999 [79]
HPV16 E7_11-20_, E7_86-93_ and PADRE	Montanide ISA 51	SC	I/II	15 recurrent or residual CxCa	No antigen-specific CTL response	2/15 SD 2/15 tumor regression after chemotherapy following vaccination	Ressing 2000 [80]
HPV16 E7_86-93_ lipopeptide and PADRE	-	SC	I	12 CxCa or vaginal cancer	Antigen-specific CTL response in 7 patients	NR	Steller 1998 [81]
HPV16 E7_12-20_, E7_86–93_ lipopeptides and PADRE	IFA	SC	I	18 high grade CIN/VIN	CTL response in 10 patients	3 CR 6 PR	Muderspach 2000 [82]
HPV16-E7_86–93_ (CIGB-228 vaccine)	Very small size proteo-liposomes (VSSP)	SC	I	7 (2 CIN 2, 5 CIN 3)	CTL response	5/7 CR and PR	Solares 2011 [83]
Four HPV16-E6 peptides	Yeast extract (Candin^®^)	IL	I	300 HSIL	Ongoing	Ongoing	NCT01653249
MAGE-A3 and HPV16 peptides	GM-CSF and Montanide ISA 51	SC	I	90 recurrent, progressive or metastatic HNSCC	Ongoing	Ongoing	NCT00257738

Abbreviations: PADRE, pan-DR T helper epitope; IFA, incomplete Freund’s adjuvant; GM-CSF, granulocyte macrophage colony-stimulating factor; SC, subcutaneous; IL, intra-lesional; CxCa, cervical cancer; CIN, cervical intraepithelial neoplasia; VIN, vulvar intraepithelial neoplasia; LSIL, low-grade squamous intraepithelial lesion; HSIL, high-grade intraepithelial lesion; HNSCC, head and neck squamous cell carcinoma; CTL, cytotoxic T lymphocyte; CR, complete response; PR, partial response; SD, stable disease; NR, no response.

### 2.4. DNA Vaccines

DNA-based vaccines have been investigated as an attractive therapeutic approach against malignancies since they are safe, can be easily produced at high purity, provide stable expression of the encoded antigen [86], and may have adjuvant functions, as plasmid DNA itself harbors unmethylated CpG motifs, which can be recognized by Toll-like receptor (TLR)-9 [87,88]. The main advantage of DNA vaccination is the production of non-live, non-replicating, non-spreading antigens that are delivered to the APCs and are capable of inducing both CTL and Th immunity, but also B-cell immunity. In addition, DNA vaccination does not induce anti-vector autoimmunity in the patient, thus multiple DNA administrations are possible without triggering an immune response against the DNA plasmid [89]. This approach may therefore be particularly useful in the context of therapeutic cancer vaccination, where repeated vaccinations are often needed for effectively boosting T cell responses.

Several preclinical and clinical DNA vaccine studies have been conducted against HPV-induced malignances. **Clinical trials** are listed in Table 3. A DNA plasmid originally named ZYC101 (MGI Pharma, formerly Zycos Inc) encoding the HLA-A2-restricted HPV16 E7_83–95_ epitope, formulated within biodegradable polymer microparticles was developed for the treatment of HPV16 infections in individuals who are HLA-A2 positive. The safety of the vaccine as well as the histological response and immune response were evaluated in a phase I clinical trial in 12 patients with anal intraepithelial neoplasia (AIN) [90]. Ten of 12 subjects mounted an antigen-specific immune response after injection with ZYC101. A phase I clinical study with ZYC101 has also been conducted in fifteen CIN2/3 patients. Eleven patients mounted HPV-specific T cell responses and five patients had complete histologic regression [91]. The next generation of this vaccine, ZYC101a, which includes HPV16 and HPV18 E6- and E7-derived CTL epitopes, was tested in a randomized, double-blind, placebo-controlled phase II study, which enrolled 127 women with CIN2/3. In this study, the proportion of subjects with resolved lesions was higher in the treatment groups, but this result did not reach statistical significance. However in a prospectively defined population of women younger than 25 years (*n* = 43), HPV clearance was significantly higher in the ZYC101a groups compared to placebo [92].

Another DNA vaccine targeting the HPV16 E7 oncoprotein, pNGVL4a-Sig/E7(detox)-Hsp70, was tested in a phase I/II clinical trial for the treatment of patients with HPV 16-positive CIN2/3 (NCT00121173). This DNA plasmid encodes a mutated form of HPV16 E7 with an abolished pRb binding site, denoted E7(detox). As DNA vaccines generate modest immunity in humans, the E7(detox) sequence was fused to a chaperone, Hsp70 from *Mycobacterium tuberculosis*, to enhance uptake by APC and MHC class I processing and presentation. The E7(detox)-Hsp70 antigen was further linked with a signal sequence, which results in secretion of E7, based on the reasoning that a secreted antigen would be more likely to gain access to professional APC than one that was expressed intracellularly. In this study, E7-specific CTL immune responses were detected in eight patients; complete histologic regression occurred in three individuals [93]. Moreover, a phase I trial of sequential heterologous prime-boost vaccination using the same DNA plasmid, pNGVL4a-Sig/E7(detox)-Hsp70 with a recombinant vaccine virus encoding a HPV16 and HPV18 E6/E7 fusion protein (TA-HPV) with or without imiquimod was evaluated in 12 patients with CIN3 (NCT00788164). Five patients showed complete histologic regression. The postvaccination immunologic changes included increased intensity of CD8^+^ infiltrates in both the stromal and epithelial compartments. These infiltrates consisted of activated effector memory T cells with potent effector functions [94].

A DNA vaccine encoding calreticulin (CRT) fused to E7(detox), pNGVL-4a-CRT/E7(detox), based on the plasmid backbone mentioned above is currently in an ongoing phase I clinical trial. The study uses the intramuscular electroporation based TriGrid^TM^ Delivery System (TDS) in combination with cyclophosphamide in patients with HPV-associated head and neck cancer (NCT01493154). The pNGVL-4a-CRT/E7(detox) DNA construct is also applied in a pilot study for treatment of HPV16-positive patients with CIN2/3, to compare immunogenicity of three different routes of administration: intradermal (ID), intramuscular (IM), and intralesional (IL) (NCT00988559).

Another candidate DNA vaccine in phase I/II clinical trials, VGX-3100, is a mixture of two plasmids that encode HPV16 and HPV18 E6/E7 antigens. Codon/RNA optimization and the addition of a highly efficient leader and Kozak sequence were incorporated into the vaccine, with the goal of increasing immune potency. An endoproteolytic cleavage site was introduced between the E6 and the E7 sequences for proper protein folding and better antigen processing. Eighteen postresection CIN2/3 subjects were enrolled in this study (NCT01304524). Fourteen patients mounted vaccine-induced HPV16 or HPV18 E6 or E7-specific cellular immune responses. These T cell responses consisted of both CTL and Th cells. The CTLs exhibited co-expression of multiple lytic markers and full cytolytic functionality [95].

While promising, none of the therapeutic DNA vaccines against HPV have yet been licensed. It is still necessary to overcome limitations and improve therapeutic DNA vaccination efficacy in clinical conditions. To this end, numerous **preclinical studies** have been conducted. Linkage of antigens to proteins capable of intercellular transport has been shown to enhance the spread of antigens encoded by DNA vaccines. Fusion genes consisting of herpes simplex virus type 1 (HSV-1) tegument protein VP22 [96] or glycoprotein gD [97] linked to HPV16 E7, have been demonstrated to increase the number of E7-expressing APCs in the lymph nodes of mice, as well as protecting against TC-1 cell challenge in murine tumor models. Strategies have also been developed to enhance antigen processing through the MHC class II pathway. Lysosomal-associated membrane protein (LAMP-1) has been employed in a DNA vaccine to enhance lysosomal antigen targeting. This vaccine resulted in increased immune responses against HPV E7 in the TC-1 tumor model [98]. To improve APC and T cell interaction, co-injection of DNA vaccines with anti-apoptotic molecules such as Bcl-xL, Bcl-2, and X-linked inhibitor of apoptosis protein (XIAP) has been employed [99]. However, administration of DNA encoding anti-apoptotic factors raises safety concerns for malignant transformation. Effective silencing of gene expression in cells by small interfering RNA (siRNA) technology targeting key proapoptotic molecules may be an attractive alternative. It has been shown that co-application of a DNA vaccine harboring HPV16 E7 with siRNA targeting the key proapoptotic proteins, Bak and Bax, can improve the survival of antigen-presenting DCs in the draining lymph nodes and enhance E7-specific CTL responses against TC-1 tumor cells in vaccinated mice [100]. Another strategy for improved DNA vaccination is based on a “shuffled” HPV16 E7 gene (HPV16 E7SH). This construct contains all potential naturally occurring CTL epitopes, but they are arranged in a different order to ensure abrogation of any oncogenic E7 properties. Immunization with HPV16 E7SH elicited strong E7-wildtype directed humoral and cellular immune responses, including tumor protection and regression in the TC-1 murine model system. Moreover, the vaccine showed *in vitro* immunogenicity in human cells, demonstrated by successful priming of antigen-specific T cells [101].

**Table 3:** Clinical studies with DNA vaccines.

Antigen/Composition	Adjuvant	Route of Injection	Phase of Study	Patient Population	Immune Response	Clinical Response	References
Plasmid encoding HPV16 E7_83-95_ (ZYC101)	-	IM	I	12 AIN	Antigen-specific responses in 10 patients	3 PR	Klencke 2002 [90]
ZYC101	-	IM	I	15 CIN 2/3	HPV-specific T cell responses in 11 patients	5 CR	Sheets 2003 [91]
Plasmid encoding HPV16 and HPV18 E6 and E7 CTL epitopes (ZYC101a/Amolimogene)	-	IM	II/III	127 CIN 2/3	Antigen-specific T cell responses in 80 patients	37 CR	Garcia 2004 [92]
Plasmid encoding mutated HPV16 E7 (E7 detox) fused to Hsp70 from *M. tuberculosis* (pNGVL4a-Sig/E7(detox)-Hsp70)	-	IM	I	15 CIN 2/3	Antigen-specific responses in 8 patients	3 CR	Trimble 2009 [93]
pNGVL4a-Sig/E7(detox)-Hsp70 (prime); TA-HPV (boost)	± Imiquimod	IM (DNA vaccine and TA-HPV) topical (Imiquimod)	I	12 CIN 3	Antigen-specific responses in 7 patients	5 CR	Maldonado 2014 [94]
Plasmid encoding HPV16 E7(detox) fused to calreticulin (CRT) (pNGVL-4a-CRT/E7(detox))	-	IM with electroporation in combination with cyclophosphamide	I	21 HNSCC	ongoing	ongoing	NCT01493154
pNGVL-4a-CRT/E7(detox)	-	ID with gene gun; IM; IL	I	39 CIN 2/3	ongoing	ongoing	NCT00988559
Mixture of two plasmids encoding HPV16 and HPV18 E6 and E7 (VGX-3100)	-	IM with electroporation	I/II	18 CIN 2/3	HPV-specific T cell responses in 14 patients	ongoing	NCT01304524 Bagarazzi 2012 [95]

Abbreviations: CTL, cytotoxic T lymphocyte; Hsp, heat shock protein; TA-HPV, recombinant vaccinia virus encoding HPV 16 and 18 E6/E7; IM, intramuscular; ID, intradermal; IL, intra-lesional; AIN, anal intraepithelial neoplasia; CIN, cervical intraepithelial neoplasia; HNSCC, head and neck squamous cell carcinoma; CR, complete response; PR, partial response.

A major advancement in DNA vaccination has been the introduction of electroporation (EP). EP involves the application of brief electric pulses to the vaccination site after intramuscular or intradermal administration of plasmid DNA. EP increases plasmid uptake and generates a local inflammatory cell infiltrate, leading to a stronger immune response to the vaccine. The safety of electroporation after DNA vaccination is comparable to that of DNA delivered without EP, with no increased risk of toxicity or integration of the plasmid DNA into the genome of the host cell [102,103]. A recent phase I clinical study using a HPV16 and HPV18 E6/E7 DNA vaccine delivered by electroporation is described above [95].

Taken together, increasing evidence suggests that DNA vaccines are valuable tools in therapeutic HPV vaccine development. EP protocols or heterologous DNA prime and viral vector-based boost regimens are applied to increase immunogenicity. As for protein/peptide vaccines (see above), combination with T regulatory cell (Treg) depletion is being evaluated to enhance clinical vaccine efficiency [104].

### 2.5. Nanoparticles

The field of nanomedicine is evolving and its promising applications have started to improve the screening and therapy options for HPV infections. However, no clinical nanoparticle vaccine studies have been conducted to date. The major advantage of nanoparticles (NPs) is that antigens and APC-activating agents (such as TLR agonists or other adjuvants) can be targeted to the same APC [105]. NPs can be used to modulate the induced immune response by modifying antigen and adjuvant characteristics, such as stability, tissue and cell targeting, and DC-activating capacity [105,106,107,108].

Different nanoparticle approaches have been used in **preclinical** therapeutic HPV vaccine studies. For instance, Tang *et al.* showed that a self-assembling NP vaccine with a HIV-1 Tat_49–57_/HPV16 E7_49–57_ fusion peptide and granulocyte-macrophage colony-stimulating factor (GM-CSF) DNA elicited potent and prolonged CTL-dependent anti-tumor immunity in mice [109]. Another vaccine, based on hepatitis B small surface antigen HBsAg(S) nanoparticles carrying short E7 epitopes (E7_11–20_ and E7_82–90_), immune-stimulatory domains of the chemokine ligand 19/macrophage inflammatory protein (CCL19/MIP-3) and interleukin 2 (IL-2), induced specific T cell responses against E7 without the need of an adjuvant in HLA-A2 (AAD) transgenic mice. Moreover, vaccination prevented the development of tumors after implantation of TC-1/A2 tumor cells [110]. Another study reported the induction of tumor-protective immunity in mice using an *E. coli*-derived recombinant HPV16 E7 that self-assembled into nano- and microparticles. TC-1 tumor-protective immunity correlated with the elicited E7-specific T cell responses, and with IgG isotype switching [111].

More recently, a novel NP-based adjuvant (“PELC”) has been used in a preclinical study. PELC contains the bioresorbable polymer poly-(ethylene glycol)-blockpoly-(lactide-co-ε-caprolactone) (PEG-b-PLACL), the surfactant Span 85 and squalene. A peptide derived from HPV16 E7, E7_49-57_, was formulated with PELC nanoparticles and CpG oligonucleotides. The PELC-based-vaccine resulted in increased numbers of interferon-γ secreting cells and antigen-specific CD8^+^ T cells and an enhanced CTL response compared with antigen formulated with PELC or CpG alone. TC-1 tumor-bearing mice received a single injection of E7_49–57_/PELC/CpG, which induced complete tumor regression [112].

Another recent report describes polymer-peptide conjugates with the ability to act as self-adjuvanting vaccines. These conjugates contained the peptide HPV16 E7_44–62_, which harbors a CTL epitope, and Th and B-cell epitopes. Several modified epitopes were generated to avoid cysteine-mediated aggregation. A single injection with these conjugates resulted in eradication of TC-1 tumors in mice, without requiring any additional adjuvant [113].

A general goal in vaccine design is **adjuvant quantity reduction**. It has been shown that targeting adjuvants to the lymph node via ultra-small polymeric NPs, which rapidly drain to the lymph node after intradermal injection, greatly enhances adjuvant efficacy at low doses. Coupling CpG oligonucleotides to NPs led to better dual-targeting of adjuvant and antigen to cross-presenting DCs compared with free adjuvant. This resulted in enhanced DC maturation and Th1 cytokine secretion, in turn driving stronger effector CTL activation with enhanced cytolytic profiles and, importantly, more powerful memory responses. Furthermore, these NPs could substantially protect mice from syngeneic tumor challenge, even 4 months after vaccination. Together, these data demonstrate that NPs can enhance vaccine efficacy at low adjuvant doses, while inducing potent and long-lived cellular immunity [114]. Also **chemical modifications** can help to further enhance NP potency. For instance, antigen conjugation to the NP surface via a disulfide bond, which can be reversibly cleaved in the reductive environment within endosomes, led to more efficient cross-presentation than antigen irreversibly conjugated to the NP surface [111].

**Liposomes and self-assembling lipo-peptides** are other attractive particle methodologies to enhance therapeutic vaccine efficiency. The lipid tail enhances epitope delivery to APCs, because lipidation increases peptide hydrophobicity and consequently permeation through biological membranes and bioavailability. It also increases chemical stability and protects against enzymatic degradation. Lipidation can target peptides to specific cells, like DCs or cancer cells, thus increasing peptide immunogenicity or antitumor efficacy [115]. An example of a liposome-based delivery system is LPD (liposome-polycation-DNA). LPD was engineered by combining cationic liposomes and polycation condensed DNA. It has been used in two **preclinical** HPV vaccination studies, using either the whole E7 protein [116] or the HPV16 E7_49–57_ peptide [117]. Both caused tumor regressions in the TC-1 tumor model. Another HPV peptide study using VacciMax^®^ liposomes has already been discussed in the *Current epitope-specific vaccine approaches* section. Several liposome formulations, among them oligomannose liposomes, have also been tested for their suitability as HPV16 DNA vaccine carriers [118].

### 2.6. Cell-Based Vaccines

Cell-based strategies for tumor immunotherapy include the use of dendritic cell vaccines or cytokine-transduced autologous tumor cells.

#### 2.6.1. DC-Based Vaccines

As DCs are the central players in eliciting antigen-specific T cell responses, they have been investigated for their applicability as vaccines themselves [119]. DC-based vaccine strategies in experimental cancer immunotherapy can be divided into DCs pulsed with peptides/proteins or DCs transduced with DNA or viral vectors encoding the target antigen. DC-based vaccines have been **clinically** tested in patients with HPV-induced cervical cancer (Table 4). In a pilot study, autologous DCs were pulsed with HPV16 or HPV18 E7 recombinant proteins and tested in fifteen late stage cervical patients. The vaccination was well tolerated and no local or systemic side effects were observed. Antigen-specific T cell immune responses were reported in 36% of patients, however, no objective clinical responses were observed [120]. Another autologous DC vaccine was pulsed with recombinant HPV16/18 E7 antigens and either IL-2 [121], or keyhole limpet hemocyanin (KLH) [122]. The latter was delivered to 10 cervical cancer patients. Antigen-specific CD8^+^ T cell immunity was observed in 80% of patients and CD4^+^ T cell responses in all vaccinated patients.

**Table 4:** Clinical studies with dendritic cell (DC)-based vaccines.

Antigen/Composition	Adjuvant	Route of Injection	Phase of Study	Patient Population	Immune Response	Clinical Response	References
Autologous DCs loaded with recombinant HPV16 or HPV18 E7	-	SC	I	15 grade IV CxCa	HPV-specific T cell responses in 4 patients	NR	Ferrara 2003 [120]
Autologous DCs loaded with recombinant HPV16 or HPV18 E7 with rhIL-2	-	SC	I	4 CxCa refractory to standard treatment	CD4^+^ T cell responses in 2 patients; antigen-specific CD8^+^ T cell response in 4 patients	NR	Santin 2006 [121]
Autologous DCs loaded with recombinant HPV16 or HPV18 E7 with KLH	-	SC	I	10 grade IB/IIA CxCa	CD4^+^ T cell response in 10 patients; antigen-specific CD8^+^ T cell response in 8 patients	Not reported	Santin 2008 [122]

Abbreviations: DCs, dendritic cells; rhIL-2, recombinant human interleukin-2; KLH, keyhole limpet hemocyanin; SC, subcutaneous; CxCa: cervical cancer; NR, no response.

Gene-transduced dendritic cell vaccines represent an attractive alternative to peptide-pulsed DCs, as HLA restriction may be bypassed, allowing presentation of peptides by all of the patient’s HLA molecules. Several **preclinical** studies of transduced DCs have been carried out in the HPV field. The use of DCs transduced with a CD40-targeted adenoviral vector carrying a mutated HPV16 E7 protein (AdE7) was evaluated in a murine tumor model. Contrary to DCs infected by untargeted Ad, DCs infected with AdE7 resulted in protection against HPV16 E7-expressing tumors. This observed protection was antigen-specific CTL dependent. Moreover, DCs cells transduced with CD40-targeted AdE7 mediated partial therapeutic immunity in mice bearing established tumors [123]. An obstacle for Ad-modified DCs is the expected lower efficacy in humans that have been previously exposed to adenoviruses. Other adenoviral vaccines (used directly and not *ex vivo* on DCs) are described in the section “Viral vector vaccines”.

To enhance DC vaccine potency, intracellular targeting of HPV16 E7 into the endosomal and lysosomal compartments was used to increase antigen presentation [124]. Alternatively, DCs transfected with siRNA targeting the IL-10 receptor have been used to block immunosuppressive processes [125]. Both approaches induced tumor regression in the TC-1 model.

Despite promising results of dendritic cell-based therapies, there are several limitations. DC vaccines require preparation of autologous DCs from each individual patient. This personalized therapy is labor-intensive and expensive, and thus may limit the large-scale production of such vaccines. In addition, only a limited amount of vaccine material is available from individual patients. Further, even when appropriately loaded with antigens and activated *in vitro*, the efficiency and function of DC-based vaccines may be significantly impaired *in vivo* by tumor-induced immunosuppression. DCs do not proliferate; consequently they undergo apoptosis after a certain time. Thus, their short half-life may limit long-lasting antigen-specific immune responses [126].

#### 2.6.2. Tumor Cell-Based Vaccines

Tumor cell-based vaccines involve systemic delivery of whole tumor cells in order to stimulate the immune system to recognize tumor-associated antigens. Tumor cells may be genetically modified *in vitro* with genes encoding co-stimulatory molecules or cytokines to increase their immunogenicity. Several HPV-positive tumor cells have been transduced with cytokine genes such as IL-2 [127,128], IL-12 [129,130], and GM-CSF [128,131]. Another study subjected TC-1 cells to photodynamic therapy (PDT), and immunized with the resulting lysate mixed with CpG oligodeoxynucleotides [132], demonstrating protective antitumor immunity in a preclinical murine model. However, administering modified malignant cells into patients raises safety concerns. Therefore, the use of tumor cells as vaccines is not considered for the treatment of early-stage or premalignant HPV-induced lesions.

### 2.7. Live Vector-Based Approaches

#### 2.7.1. Viral Vector Vaccines

Advances in molecular virology have allowed the genetic manipulation of viruses, which has opened new opportunities for vaccine development. Not only can viruses be attenuated far more rapidly by modifying parts of their genome, but they can also be employed as vaccine carriers, harboring a sequence of a target antigen. Recombinant viral vector vaccines have several advantages. First, they can be developed rapidly. They induce a full spectrum of immune responses including antibodies and antigen-specific T cells that are crucial for control of intracellular pathogens and cancer [133]. The main disadvantages for all viral vector-based vaccines are pre-existing immunity and the induction of neutralizing antibodies against the viral vector, which impairs their ability to elicit potent primary or secondary responses, respectively. This can be overcome by the use of viruses that do not circulate in humans, such as viruses that preferentially infect other species, or by switching serotypes for booster immunizations. Further limitations of viral vector-based vaccines are related to safety concerns, high production costs and their stability [134]. Many viral species have been evaluated as recombinant vectors for vaccines against HPV, including vaccinia viruses, adenoviruses (AdV), alphaviruses (such as Semliki forest virus and Sindbis virus) and lentiviruses.

Vaccinia virus (VACV), a member of the poxvirus family, is a promising vector due to its well-characterized safety profile; it is generally safe, except in individuals with immunosuppression, cardiac disease, or atopic dermatitis [135]. VACV is known to infect a wide range of cells, it replicates exclusively within the host cell cytoplasm and there is no evidence of viral genome integration into the host genome [136]. The first HPV vaccine based on the recombinant vaccinia virus strain Wyeth is called TA-HPV, encoding E6/E7 fusion proteins of HPV16 and HPV18. The E7 sequence was mutated to abrogate pRb binding capacity. **Clinical studies** with TA-HPV are summarized in Table 5. The use of TA-HPV in a clinical trial testing a heterologous DNA prime/viral vector boost vaccination regimen is described in the “*DNA vaccines*” section, and in heterologous protein/viral vector prime/boost regimens in the “*Protein-based vaccines*” section. A phase I/II clinical study using TA-HPV was conducted in eight patients with late stage cervical cancer. After a single dose vaccination, HPV-specific CTL responses were observed in one patient [137]. Another phase I clinical trial assessed the safety and immunological effects of two vaccinations with TA-HPV in a group of 29 stage IB/IIA cervical carcinoma patients. Vaccination was well tolerated in all patients. HPV-specific CTL responses were found in four patients and eight patients developed HPV-specific antibody responses [138]. The subsequent phase II study enrolled twelve patients with high-grade HPV16-positive VIN or vaginal intraepithelial neoplasia (VAIN). HPV-specific T cell responses were found in 6 patients. Five of 12 patients showed a lesion reduction of at least 50% and one patient experienced complete regression [139]. Another 18 patients with HPV16-positive high-grade VIN were enrolled in a phase II clinical study to assess the immunological and clinical responses after vaccination with TA-HPV. In this study, increased HPV16-specific immune responses were mounted in 13 patients and eight patients demonstrated regression in lesion diameter of at least 50% [140].

The MVA-E2 viral-based vaccine is a Modified Vaccinia Ankara virus, an attenuated replicon-deficient vaccinia strain, expressing the E2 protein [141,142,143]. A phase I/II trial was performed in 34 subjects with CIN 2/3. Complete histologic regression of high-grade lesions was observed in 20 patients. Eleven patients had a 50% reduction in lesion size. All vaccinated patients developed HPV-specific antibodies, and generated specific cytotoxic responses against HPV-transformed cells. Control patients treated with conization to remove lesions and not vaccinated with MVA-E2 did not develop specific cytotoxic activity against cancer cells nor did they eliminate HPV. However, no placebo control group was enrolled in this study [142]. A second MVA-based vaccine for HPV-induced lesion treatment is named MVA-HPV-IL2 or TG4001 (Transgene SA, Strasbourg, France). The MVA vector was engineered to express mutation-inactivated HPV16 E6 and E7 proteins as well as IL-2 as an adjuvant to enhance antigen-specific immune responses. In a phase II study, 21 patients with HPV16-related CIN2/3 were enrolled. Immunization with TG4001 was associated with low systemic side effects and seven patients demonstrated complete histologic regression of high-grade lesions. CIN2/3 lesions regressed spontaneously in 20% of patients [144]. Another phase II clinical trial (randomized *vs.* placebo) targets 209 patients with single or multiple high-risk HPV infection and includes immunomonitoring studies (NCT01022346).

Recombinant adenoviral (rAd) vectors have also been tested extensively as vaccine carriers. For vaccination, most vectors are deleted in the adenoviral genes E1 or E1/E3. Initially, most vaccines were based on adenovirus type 5 (Ad5). The rationale for use of rAd vaccines includes genome stability, ease of manipulation and natural tropism for mucosal sites. Ads infect a broad spectrum of cells, including DCs, allowing for efficient antigen presentation, and can therefore also prime robust cell-mediated immune responses [145,146]. The major limitation of the use of Ad5-based vectors is the high prevalence of pre-existing humoral responses in the human population. **Preclinical studies** in a murine model have shown that immunization with a rAd5 vector expressing HPV16 E6/E7 (Ad5 E6/E7) can induce HPV antigen-specific immune responses and can prevent development of HPV16-positive tumors. However, no therapeutic effects of Ad5 E6/E7 were demonstrated [147].

**Table 5:** Clinical studies with viral vector vaccines.

Antigen/Composition	Adjuvant	Route of Injection	Phase of Study	Patient Population	Immune Response	Clinical Response	References
Recombinant vaccinia virus strain Wyeth encoding a HPV16 and HPV18 E6/E7 fusion protein (TA-HPV)	-	Dermal scarification	I/II	8 advanced CxCa	HPV-specific antibody responses in 3 patients; HPV-specific CTL response in 1 patient	Not reported	Borysiewicz 1996 [137]
TA-HPV	-	Dermal scarification	I	29 grade IB/IIA CxCa	HPV-specific antibody response in 8 patients; HPV-specific CTL responses in 4 patient	Not reported	Kaufmann 2002 [138]
TA-HPV	-	Dermal scarification	II	12 VIN	HPV-specific T cell responses in 6 patients	1 CR 5 PR	Baldwin 2003 [139]
TA-HPV	-	Dermal scarification	II	18 VIN 2/3	HPV-specific immune responses in 13 patients	8 PR	Davidson 2003 [140]
Modified vaccinia virus Ankara encoding BPV E2(MVA-E2)	-	Intra-cervical	I/II	36 CIN 1–3	HPV-specific CTL responses in all patients	34 CR	Corona 2004 [141]
MVA-E2	-	Intra-cervical	I/II	34 CIN 2/3	HPV-specific CTL responses in all patients	20 CR 11 PR	García-Hernández 2006 [142]
MVA-E2	-	Intra-urethral	I/II	30 male patients with intraurethral flat condyloma	HPV-specific cytotoxic response in all patients	28 CR	Albarran 2007 [143]
MVA encoding HPV16 E6 and E7 and rhIL-2 (MVA-HPV-IL2)	-	SC	II	21 CIN 2/3	Not reported	7 CR	Brun 2011 [144]
MVA-HPV-IL2	-	SC	II	209 CIN 2/3	Not reported	Not reported	NCT01022346

Abbreviations: BPV, bovine papillomavirus; rhIL-2, recombinant human interleukin-2; SC, subcutaneous; CxCa, cervical cancer; CIN, cervical intraepithelial neoplasia; VIN, vulvar intraepithelial neoplasia; CTL, cytotoxic T lymphocyte; CR, complete response; PR, partial response

Alphaviruses have received considerable attention for use as vaccination vectors. The main advantage of alphaviruses is transient but high-level cytoplasmic expression of a heterologous gene. The alphaviral vectors are also called ‘replicons’ because of the self-replicating nature of the alphavirus genome. Since an RNA virus vector cannot integrate into chromosomal DNA, the risk of cellular transformation is negligible. Currently, vectors derived mainly from Semliki Forest virus (SFV), Sindbis virus (SIN) and Venezuelan equine encephalitis virus (VEE) are intensively being developed for therapeutic approaches [148], but have not reached the clinical trial stage in the HPV field. Recombinant SFV encoding a fusion protein of HPV16 E6 and E7 (SFVeE6-E7) was able to elicit strong and long-lasting antigen-specific immune responses and also eradicated established tumors in mice [149,150,151]. A homologous booster immunization increased CTL activity and induction of protective central memory CTL responses [149,150] compared to a single priming immunization with recombinant SFV. To enhance the efficacy of the SFVeE6-E7 vaccine, the adjuvant effect of IL-12 was utilized by co-administration of SFVeE6-E7 with a SFV vector harboring IL-12 [152]. SIN vectors carrying HPV16 E7 have also been developed to generate immunity against HPV16-induced tumors. Vaccination with SIN-E7 has shown poor immunogenicity. The potency of this sindbis virus-based vector was improved by enhancing uptake, processing and presentation of E7 by DCs. This was achieved by fusion to the chaperone Hsp70 [153], calreticulin [154], or the HSV-1 VP22 protein [155], or by targeting E7 to the endosomal/lysosomal compartments by fusion with LAMP-1 [156]. Vaccination of mice with replication-defective VEE replicon particles carrying HPV16 E7 RNA (E7-VRP) induced CTL responses and eradicated established tumors in 67% of tumor-bearing mice [157]. Another study found that VEE replicon particles encoding a fusion protein of mutated HPV16 E6 and E7 elicited E7-specific CTL immunity and eradicated established tumors in 90% of tumor-bearing mice [158].

Lentiviruses have emerged as a very potent class of viral vectors for antitumor immunotherapy due to their ability to transduce a variety of different dividing and non-dividing cell types, including tumor cells and DCs. Immunizations with lentiviral vectors (LV) have demonstrated the induction of potent antigen-specific T cell responses that were capable of controlling tumor growth [159]. The main obstacles toward the use of recombinant lentiviruses as vector-based therapies are serious safety concerns due to the potential for malignant transformation of target cells following insertional mutagenesis. However, integrase defective lentiviral vectors (IDLV) have been engineered to minimize this risk [160]. A therapeutic vaccine against HPV16 based on IDLV carrying a mutated form of HPV16 E7 fused to calrecticulin (IDLV-CRT/E7) was evaluated in the TC-1 tumor model. Vaccination with IDLV-CRT/E7 elicited both cellular and humoral tumor-specific immune responses. A single intramuscular immunization, without adjuvants, chemotherapy or booster vaccinations, was able to induce long-lasting high levels of antigen-specific polyfunctional CTL responses. These were sufficient to control tumor growth in an early-stage tumor model and to completely eradicate tumors in tumor-bearing mice [161].

#### 2.7.2. Bacterial Vector Vaccines

Another strategy for tumor-targeted immunotherapy is the use of bacterial vectors. Attenuated strains of *Listeria monocytogenes* are currently being evaluated for this purpose. *L. monocytogenes* is a Gram-positive intracellular bacterium that infects macrophages. Due to secretion of lysteriolysin O (LLO), the bacterium can escape phagosomal lysis and replicate in the cytoplasm. Therefore, *L. monocytogenes* can carry foreign antigens into both the MHC class I and II pathways. *L. monocytogenes* immunotherapy stimulates both innate and cell-mediated adaptive immunity [162].

A **preclinical** study has demonstrated that immunization with recombinant *L. monocytogenes* expressing HPV16 E7 (Lm-E7) can induce measurable antigen-specific CTL responses. However, the ability to eliminate established TC-1 tumors was only partial [163]. To enhance Listeria-based vaccine potency, the Listeria protein LLO (non-hemolytic fragment of LLO) was used as antigen fusion partner. Vaccination studies with this construct have shown antigen-specific CTL responses and also regression of HPV16-expressing tumors in tumor-bearing mice [163]. **Clinical studies** with bacterial vector vaccines are listed in Table 6. A phase I clinical trial using recombinant *L. monocytogenes* Lm-LLO-E7 (ADXS11-001) was conducted in 15 late stage metastatic cervical cancer patients who had failed prior chemotherapy, radiotherapy or surgery [164]. In this study, 4 of 13 evaluable patients experienced a reduction of their tumor load. ADXS11-001 is currently being evaluated in a multicenter phase II clinical trial in 67 patients with persistent or recurrent cervical cancer (NCT01266460), as well as in a randomized, single-blind, placebo-controlled phase II study in a cohort of 120 patients with CIN2/3 (NCT01116245). Furthermore, a phase I/II safety and efficacy trial of ADXS11-001 has been initiated for treatment of HPV16-positive oropharyngeal cancer (NCT01598792).

**Table 6:** Clinical studies with bacterial vector vaccines.

Antigen/Composition	Adjuvant	Route of Injection	Phase of Study	Patient Population	Immune Response	Clinical Response	References
Live attenuated *L. monocytogenes* secreting HPV16 E7-LLO fusion protein (ADXS11-001)	-	IV	I	15 high-grade CxCa	HPV-specific T cell response in 1 patient	4 CR	Maciag 2009 [164]
ADXS11-001	-	IV	II	67 high-grade CxCa	ongoing	ongoing	NCT01266460
ADXS11-001	-	IV	II	120 CIN 2/3	ongoing	ongoing	NCT01116245
ADXS11-001	-	IV	I/II	36 HPV16^+^ oropharyngeal carcinomas	ongoing	ongoing	NCT01598792

Abbreviations: LLO, lysteriolysin O; IV, intravenous; CxCa, cervical cancer; CIN, cervical intraepithelial neoplasia; CR: complete response.

## 3. Adjuvants and Vaccine Delivery Technologies

Adjuvants are compounds that enhance the magnitude, breadth, quality and duration of specific immune responses to antigens. Addition of adjuvants to vaccines may reduce the amount of antigen and/or the number of immunizations required to achieve the desired immune responses [165]. The adjuvant concept is more than 80 years old, with the first and yet most commonly used adjuvant in human vaccines, an aluminum salt (aluminum potassium sulphate, also known as alum), appearing in the 1920s. Aluminum salts have been sufficient to induce an adequate, mainly humoral, immune response for most of the licensed vaccines. However, many modern vaccines, including cancer vaccines, consist of highly purified antigens with reduced immunogenicity and therefore require stronger adjuvants [166].

For an efficient therapeutic cancer vaccine, it is essential to induce, expand and maintain a tumor associated antigen (TAA)-specific CD8^+^ T cell population. Due to both central and peripheral tolerance mechanisms, TAAs are poorly immunogenic. When administered alone without appropriate immunostimulatory signals, they often elicit immunological tolerance by specific T cell anergy or the induction of regulatory T cells. Key to inducing a robust cytotoxic immune response is the potent triggering of innate immunity, leading to the recruitment, activation, and maturation of APCs such as DCs. Adjuvants eliciting a potent Th1 pro-inflammatory stimulus are of central importance to the development of effective therapeutic cancer vaccination strategies [165].

Several adjuvants have been developed to increase the potency of cancer vaccines. These adjuvants have ranged from general immune stimulants such as the live-attenuated tuberculosis vaccine *Bacille Calmette-Guérin* (BCG) to molecularly defined compositions that trigger specific receptors (see below). Other non-specific adjuvants, such as Hsp’s and keyhole limpet hemocyanin (KLH) have been long used as conjugates in recombinant protein or peptide-based vaccines. The adjuvant effects of KLH have been attributed to its repetitive carbohydrate residues, while it also provides Th epitopes [167]. Hsp’s such as Hsp70 and Gp96 enhance vaccine potency by chaperoning antigenic peptides to MHC class I molecules at the cell surface for presentation to lymphocytes; while antigen-independent activation of innate immunity has been reported as well [168]. Another approach is to directly supply the immune system with the cytokines that would be produced in response to activation of innate immunity. Cytokines are applied as recombinant proteins, as fusion partners with selected TAAs, co-expressed with TAAs in DNA-based cancer vaccines, or expressed in transduced whole tumor cell based vaccines (c.f. sections above).

The design of adjuvants has evolved with the understanding that the interaction of conserved pathogen-associated molecular patterns (PAMPs) with specific pattern recognition receptors (PRRs, such as Toll-like receptors, TLRs) leads to activation of NF-κB and IRF-3, and subsequent expression of pro-inflammatory cytokines. This forms the basis of innate immunity, which in turn triggers adaptive immune responses [169]. The addition of various TLR agonists to vaccine formulations has been a significant step forward in cancer vaccine adjuvant development. These include ligands of TLR-3 (poly I:C), TLR-4 (monophosphoryl lipid A; MPLA), TLR-5 (flagellin), TLR-7 (imiquimod), TLR-7/8 (resiquimod), and TLR-9 (CpG) [170,171,172]. Either individually or in combinations, these TLR agonists have been shown to significantly enhance vaccine potency [173].

The TLR-targeted adjuvants are typically formulated as microparticles/nanoparticles (e.g., oil-in-water emulsions, saponin-containing formulations including QS-21, immunostimulating complexes (ISCOMS and ISCOMATRIX™) or liposomes, together with selected antigens [174]. For example, MPLA is a component of Cervarix^®^, the prophylactic HPV16/18 vaccine developed by GlaxoSmithKline Biologicals (GSK) [175]. A synthetic TLR-4 agonist known as glucopyranosyl lipid A (GLA) has been evaluated clinically as an adjuvant for a seasonal influenza vaccine, and is also being developed as a cancer vaccine adjuvant [176]. Preclinical and early clinical data support the use of TLR9 agonists (CpGs) as vaccine adjuvants, where they can enhance both the humoral and cellular responses to diverse antigens [177]. Furthermore, combination of adjuvants has shown promising results in preclinical studies of therapeutic HPV vaccines [48,178].

There are several promising ongoing clinical studies that utilize defined molecular adjuvants (reviewed in [179]). GSK is currently conducting a phase III clinical study to evaluate the efficacy of its melanoma antigen epitope-3 (MAGE-A3) antigen-specific cancer immunotherapy (ASCI) in subjects with non-small cell lung cancer (NSCLC) [180]. This cancer vaccine consists of recombinant MAGE-A3 protein formulated in saponin-containing QS-21 liposomes containing the TLR-4 and TLR-9 agonists, MPL and CpG oligodeoxynucleotides.

In recent clinical trials of therapeutic HPV vaccine candidates, different adjuvants have been used, like the squalene based water in oil emulsion Montanide ISA 51 [49,50,51,52,53,54,80], GM-CSF with Montanide ISA 51 (NCT00257738), AS02A [41], AS02B [35], imiquimod (PC10VAC01 and -02, and [30,94]), or ISCOMATRIX™ [34]. All adjuvants used in HPV trials are indicated in the respective tables of this review.

## 4. Outlook

### 4.1. Combination Therapies

A lesson learned from all cancer immunotherapy studies to date is that the presence of tumor-specific cytotoxic cells is not sufficient for therapeutic success in a tumor-bearing host. It is equally important to overcome the immune-suppressive mechanisms acting in the tumor microenvironment. Effective treatment regimens require the use of different therapies having distinct mechanisms of action. Some established treatments synergize with immunotherapy. For example, some chemotherapeutic agents have been shown to enhance cross-presentation, thus augmenting tumor-specific adaptive immune response [181]. Cyclophosphamide at low doses has been shown to inhibit regulatory T cells, and is being evaluated in combination with therapeutic immunization regimens in early-phase clinical studies [182]. Other compounds have been specifically developed to overcome tumor immune suppression. This has been termed “immune checkpoint blockade”, with the model agent being the anti-CTLA4 monoclonal antibody (mAb) ipilimumab. A pioneering clinical trial showed that ipilimumab, with or without a gp100 peptide vaccine, improved overall survival in patients with previously treated metastatic melanoma [183]. Additional mAbs that block another checkpoint, the PD-L1/PD-1 interaction, have been shown to significantly enhance vaccine potency and to overcome T cell “exhaustion,” and are currently being evaluated as single agents in both cancer and infectious disease clinical settings [184,185].

### 4.2. Novel Materials

New polymerization technologies may influence future vaccine design. For instance, macroporous polymer matrices have been used as carriers for cancer vaccines, and were capable of directing the trafficking and activation of DCs *in vivo* by precisely controlling the presentation of different adjuvants. These were mainly TLR ligands [186,187]. When applied as therapeutic cancer vaccines (B16-F10-melanoma-tumor lysates were loaded into PLG scaffolds), these matrices led to CTL-mediated eradication of melanomas in mice [186,188].

### 4.3. Vaccination Routes

The route of vaccine administration significantly influences the magnitude and compartmentalization of the immune response. For example, a preclinical study showed that the growth of an orthotopic HPV-associated head and a neck tumor was inhibited when a cancer vaccine (STxB-E7_43–57_, consisting of the E7_43–57_ peptide coupled to the nontoxic B subunit of Shiga toxin) was delivered by the intranasal route but not the intramuscular route. The intranasally administered vaccine elicited antigen specific CTLs homing to the mucosa and thus to the tumors [189]. However, a recent clinical trial showed that intramuscular therapeutic HPV16 DNA vaccination (TA-HPV, discussed in Section *Viral Vector Vaccines*) induces T cell responses that localize to mucosal lesions [94]. The opposing findings of these two studies may be due to the different nature of the vaccines used (peptide *vs.* DNA), but also stress that murine *in vivo* data always needs to be interpreted with caution.

### 4.4. Advanced Mouse Models

Performing immunization experiments in novel humanized mouse models may yield better predictions for the outcome in humans [190]. One such model is the HLA-A2.1-/HLA-DR1-transgenic H-2 class I-/class II-knockout mouse [191]. The immunological potential of this model was evaluated in response to a hepatitis B DNA vaccine. Every mouse immunized developed hepatitis B virus-specific antibodies, HLA-DR1-restricted Th, and HLA-A2.1-restricted CTL responses directed at the same immunodominant epitopes as those identified in naturally infected or vaccinated humans [191]. These mice represent a unique *in vivo* experimental model for human immune function studies without any interference of mouse MHC responses, which previously complicated the prediction of human responses. Still, even data obtained from advanced mouse models should be carefully interpreted, since beside the humanized parts, these mice still have a murine immune system that largely differs from the human one [191]. In the end, well designed proof-of-concept clinical trials are the only way to evaluate the efficacy of therapeutic vaccines.

### 4.5. Systems Biology and Lab-on-a-Chip Techniques

Most currently used adjuvants have been empirically discovered with little knowledge about their mechanism of action or possible side effects. Recent advances in vaccinology have paved the way for more rational methods of vaccine development. These new technologies, such as reverse vaccinology, structural vaccinology, systems vaccinology and systems immunogenetics, are paving the way for the design and development of so-called “third generation” vaccines [192,193].

**Systems vaccinology** is an emerging new methodology; it refers to applying tools from systems biology to vaccine studies [194,195,196,197]. The tools of systems biology consist of a number of high-throughput technologies, including DNA microarrays, protein arrays, deep sequencing and mass spectrometry [198]. They allow system-wide unbiased molecular measurements, which can then be used to reconstruct the events during an immune response. For example, about a week after the administration of influenza vaccines, antibody producing plasma blasts increase in the blood and their gene expression pattern will appear in the transcriptomic profile measured from peripheral blood [199]. Published studies have used systems biology approaches to identify molecular networks that shape immunity in response to vaccination in humans [194,195,200]. Analyses of the immune response to the vaccine against yellow fever virus (YF-17D) have provided proof of the concept that molecular signatures in the blood of humans, induced within a few days after vaccination, can be used to predict the magnitude of the later immune responses to a vaccine and are beginning to yield insights about the nature of the innate and adaptive responses to vaccination [201,202]. Subsequently, systems biology approaches have been extended to identify predictive signatures of vaccines against influenza virus and are being used to study immune responses to other vaccines [203,204,205]. The new field of systems vaccinology can address the mechanisms that control immune responses to vaccination and identify predictors of vaccine efficacy [197].

**Systems immunogenetics** provides another powerful and robust framework for vaccine discovery, development, and delivery. Mechanistic studies can reveal new vaccine targets or “rescue” previously discarded candidates by elucidating how genetic variation can influence innate and adaptive immune responses to vaccines. This can provide signatures to allow early patient stratification related to vaccine failure and adverse events. Emulating personalized medicine approaches from oncology and other disciplines will allow for more ‘precise’ vaccine development for subsets of patients relative to their immune phenotype and genomic architecture [206]. For instance, a study combining genetic, transcriptional, and immunologic data in people having received a seasonal influenza vaccine has identified 20 genes exhibiting a transcriptional response to vaccination, significant genotype effects on gene expression, and correlation between the transcriptional and antibody responses. That study showed that variation of genes involved in membrane trafficking and antigen processing significantly influences the human response to influenza vaccination [207]. Another study identified HLA-restricted recognition of measles virus epitopes with quantifiable impacts on immunity that appeared to be overcome by additional dosing regiments [208]. This suggests that there is tremendous potential for systems immunogenetics guided stratification and individualized vaccine delivery [209].

**Lab-on-a-chip approaches** have been developed to identify antigen-specific responses *ex vivo* from 10^4^–10^5^ single cells of blood or mucosal tissues using dense arrays of subnanoliter wells. These combine on-chip imaging cytometry with a technique for capturing secreted proteins—called microengraving—to enumerate antigen-specific responses by single T cells in a manner comparable to conventional assays such as ELISpot and intracellular cytokine staining. Unlike those assays, however, the individual cells identified can be recovered readily by micromanipulation for further characterization *in vitro*. Applying this method to assess HIV-specific T cell responses demonstrated that it is possible to establish clonal CD8^+^ T-cell lines that represent the most abundant specificities present in the circulation. 100- to 1000-fold fewer cells were required than in traditional approaches [210]. The microengraving-based approach would be ideal in monitoring antigen-specific T cell responses in situations when clinical samples contain low numbers of immune cells. As this is the case in mucosal tissues, the technique could be highly useful for studying infiltrating T cells in HPV-affected tissues.

### 4.6. Immunomonitoring

Finally, rational clinical development of any therapeutic vaccine should be accompanied and guided by a comprehensive biomarker program, including T cell response monitoring. Immunomonitoring studies reflect relevant mechanisms of immune induction and tolerance, and assist in defining patient subpopulations with a higher prospect of response to vaccination [192,211].

## 5. Conclusions

Taken together, HPV-mediated tumors are an ideal scenario for proof-of-concept studies of therapeutic cancer vaccination. Many promising approaches have been developed, with several having reached advanced clinical trial stages. As new insights from vaccinology research are being incorporated, an efficient therapeutic HPV vaccine, at least for premalignant lesions, seems to be within reach in the near future.
